# Disminución de la debilidad muscular adquirida en una unidad de cuidados intensivos con la implementación de un protocolo multicomponente: ensayo clínico cuasiexperimental

**DOI:** 10.7705/biomedica.6947

**Published:** 2023-12-01

**Authors:** Nelson Darío Giraldo, Carlos Carvajal, Fabián Muñoz, María del Pilar Restrepo, Michael Andrés García, Juan Miguel Arias, José Leonardo Mojica, Juan Carlos Torres, Álex García, Diego Muñoz, Francia Cecilia Rodríguez, Jorge Arias, Lina María Mejía, Gisela De La Rosa

**Affiliations:** 1 Departamento de Cuidado Crítico del Adulto, Hospital Pablo Tobón Uribe, Medellín, Colombia Hospital Pablo Tobón Uribe Medellín Colombia; 2 Unidad de Rehabilitación, Hospital Pablo Tobón Uribe, Medellín, Colombia Hospital Pablo Tobón Uribe Medellín Colombia; 3 Sección Farmacia Clínica, Hospital Pablo Tobón Uribe, Medellín, Colombia Hospital Pablo Tobón Uribe Medellín Colombia; 4 Facultad de Medicina, Universidad CES, Medellín, Colombia Universidad CES Universidad CES Medellín Colombia; 5 Sección de Anestesiología y Reanimación, Universidad de Antioquia, Medellín, Colombia Universidad de Antioquia Universidad de Antioquia Medellín Colombia

**Keywords:** unidad de cuidados intensivos, enfermedad crítica, delirio, Intensive care units, critical illness, delirium

## Abstract

**Introducción.:**

La debilidad adquirida en las unidades de cuidados intensivos es una complicación frecuente de los pacientes con enfermedades críticas, que puede tener un impacto negativo en su pronóstico a corto y a largo plazo.

**Objetivos.:**

Evaluar si la utilización de un protocolo multicomponente, que incluye movilidad activa temprana, manejo efectivo del dolor, reducción de la sedación, medidas no farmacológicas para prevenir el *delirium,* estimulación cognitiva y apoyo familiar, puede disminuir la incidencia de debilidad adquirida en las unidades de cuidados intensivos al momento del egreso del paciente.

**Materiales y métodos.:**

Se trata de un ensayo clínico, no aleatorizado, en dos unidades de cuidados intensivos mixtas de un hospital de tercer nivel. Los participantes fueron pacientes mayores de 14 años con ventilación mecánica invasiva por más de 48 horas. Se aplicó como intervención un protocolo multicomponente y como control se utilizó el cuidado usual o estándar.

**Resultados.:**

Ingresaron 188 pacientes al estudio, 82 al grupo de intervención y 106 al grupo control. La tasa de debilidad adquirida en las unidades de cuidados intensivos al egreso de la unidad fue significativamente menor en el grupo de intervención (41,3 % versus 78,9 %, p<0,00001). La mediana del puntaje de movilidad al momento del alta de la unidad de cuidados intensivos fue mayor en el grupo de intervención (3,5 versus 2, p<0,0138). No se encontraron diferencias estadísticamente significativas en las medianas de días libres de respiración mecánica asistida, ni de unidad de cuidados intensivos al día 28, tampoco en la tasa de mortalidad general al egreso del hospital (18 versus 15 días, p<0,49; 18,2 % versus 27,3 %, p<0,167).

**Conclusiones.:**

Un protocolo multicomponente que incluía movilidad activa temprana tuvo un impacto significativo en la reducción de la debilidad adquirida en las unidades de cuidados intensivos al egreso en comparación con el cuidado estándar.

## Abstract (en)

**Introduction.:**

Intensive care unit-acquired weakness is a frequent complication that affects the prognosis of critical illness during hospital stay and after hospital discharge.

**Objectives.:**

To determine if a multicomponent protocol of early active mobility involving adequate pain control, non-sedation, non-pharmacologic delirium prevention, cognitive stimulation, and family support, reduces intensive care unit-acquired weakness at the moment of discharge.

**Materials and methods.:**

We carried out a non-randomized clinical trial in two mixed intensive care units in a high-complexity hospital, including patients over 14 years old with invasive mechanical ventilation for more than 48 hours. We compared the intervention -the multicomponent protocol- during intensive care hospitalization versus the standard care.

**Results.:**

We analyzed 82 patients in the intervention group and 106 in the control group. Muscle weakness acquired in the intensive care unit at the moment of discharge was less frequent in the intervention group (41.3% versus 78.9%, p<0.00001). The mobility score at intensive unit care discharge was better in the intervention group (median = 3.5 versus 2, p < 0.0138). There were no statistically significant differences in the invasive mechanical ventilation-free days at day 28 (18 versus 15 days, p<0.49), and neither in the mortality (18.2 versus 27.3%, p<0.167).

**Conclusion.:**

A multi-component protocol of early active mobility significantly reduces intensive care unit-acquired muscle weakness at the moment of discharge.

La debilidad adquirida en la unidad de cuidados intensivos es un síndrome de debilidad muscular simétrica que afecta comúnmente a pacientes que han sobrevivido a enfermedades críticas. Se estima que su prevalencia oscila entre el 26 y el 65 % en pacientes que han recibido ventilación mecánica invasiva por un período mayor a cuatro días ^1^^,^^2^. La presencia de este tipo de debilidad está asociada con mal pronóstico durante la hospitalización y después del alta, aumento en la duración de la respiración mecánica asistida, mayor mortalidad hospitalaria, deterioro cognitivo a corto y mediano plazo, necesidad de institucionalización después del alta y disminución de la calidad de vida hasta dos años después de haber sufrido un síndrome de dificultad respiratoria aguda ^3^^,^^4^. Los factores de riesgo identificados para la debilidad adquirida en la unidad de cuidados intensivos incluyen edad avanzada, inmovilidad, sedación, falla orgánica múltiple, hiperglucemia y ventilación mecánica invasiva ^1^^,^^5^^,^^6^.

La *Society of Critical Care Medicine* (SCCM) ha recomendado la aplicación de un paquete de medidas llamado *The ICU Liberation ABDCE Bundle.* Este paquete consiste en intervenciones basadas en la evidencia para reducir la mortalidad a corto y largo plazo, disminuir la ventilación mecánica invasiva prolongada, mejorar la pérdida funcional y aminorar la necesidad de institucionalización ^7^. A pesar de su eficacia demostrada, su implementación a gran escala sigue siendo limitada ^8^.

Por lo tanto, en este estudio se propone la implementación de un modelo de atención multicomponente y protocolizado basado en el *ABCDEF Bundle* de la SCCA, que incluya la movilidad activa temprana, el uso limitado de sedantes, adecuada analgesia, medidas no farmacológicas para prevenir el *delirium,* estimulación cognitiva y compromiso-empoderamiento de los familiares. Con esta propuesta probablemente se pueda lograr un impacto positivo en la disminución de la incidencia de debilidad adquirida en las unidades de cuidados intensivos al egreso de esta.

## Materiales y métodos

Se trata de un ensayo clínico no aleatorizado, comparativo de dos modelos de atención detallados en el anexo 1. Se llevó a cabo en dos unidades mixtas de cuidados intensivos de un hospital académico de tercer nivel con 476 camas de hospitalización y tres unidades de cuidados intensivos, dos de 14 camas y una de 12 camas.

La duración del estudio fue desde el 1 **°** de diciembre del 2018 hasta el 31 de mayo del 2019, con seguimiento hasta el egreso del hospital. Las fisioterapeutas y el personal de enfermería no cambiaron de unidad durante el periodo de estudio. No fue posible limitar la movilidad de los médicos entre las dos unidades de cuidados intensivos del ensayo.

La intervención consistió en la aplicación de un modelo multicomponente y protocolizado durante la estancia en la unidad y consistió en rondas diarias de lunes a viernes por un grupo multiprofesional (médico intensivista no tratante, intensivista tratante, fisiatra, fisioterapeuta, terapeuta respiratorio, químico farmacéutico y enfermería), donde se verificaba:

1. control adecuado del dolor;
2. pacientes despiertos con RASS de 0 a -1 (9) y evasión del midazolam en infusión continua;
3. medidas no farmacológicas para prevenir el *delirium* (estimulación cognitiva, orientación espacio-temporal por parte del personal tratante y la familia);
4. iniciación de la movilidad activa temprana en las primeras 48 horas del ingreso del paciente a la unidad de cuidados intensivos y su aplicación guiada por un protocolo (la fisioterapeuta estaba a cargo de 12 pacientes), y
5. participación de los familiares en el cuidado del paciente con acompañamiento las 24 horas del día.

El control del estudio fue el manejo estándar que se estaba aplicando en todas las unidades de cuidados intensivos antes de julio de 2018 y consistió en:

1. Evaluación, prevención y tratamiento del dolor, por medio de la aplicación de una escala de dolor y un protocolo de manejo adoptado por el hospital tiempo atrás.
2. Sedación a los pacientes con ventilación mecánica invasiva con midazolam en infusión continua. El nivel de sedación, cuantificado por la escala RASS, era decidido por el intensivista tratante.
3. Tamización de *delirium* con la aplicación de las escalas UCI-CAM (durante la respiración mecánica asistida) o CAM-S (posterior al retiro de la respiración mecánica asistida) por parte de enfermería, lo cual fue implementado años atrás. La confirmación del diagnóstico de *delirium* fue realizado por psiquiatría y se manejó con medicamentos antipsicóticos.
4. La fisioterapia o movilidad se iniciaba cuando el intensivista tratante decidiera. El tipo de ejercicio y movilización eran definidos por la fisioterapeuta sin seguir ningún protocolo. La fisioterapeuta estaba a cargo de dos UCI (28 pacientes);
5. Los familiares de los pacientes no se comprometían con el cuidado del paciente y podían permanecer 16 horas acompañándolo.

La población incluyó pacientes consecutivos, mayores de 14 años, hospitalizados en las unidades de cuidados intensivos con ventilación mecánica invasiva por más de 48 horas. Se excluyeron pacientes con dificultad para comunicarse, secuelas neurológicas graves, demencia previa y aquellos que egresaron en las primeras 48 horas o se esperaba su fallecimiento en las próximas 24 horas.

La investigación fue aprobada por el comité de ética del hospital. La recolección de los datos se llevó a cabo en un formulario diseñado previamente. La implementación del modelo multicomponente y protocolizado en la unidad de cuidados intensivos del grupo de intervención fue liderada por un equipo multiprofesional y comenzó con la educación del personal en los cinco meses previos.

*Variables:* se registraron variables demográficas como la edad y el sexo, así como el puntaje APACHE II y el índice de comorbilidades de Charlson. Se registró el diagnóstico de ingreso y se llevó a cabo un tamizaje del *delirium* en la unidad de cuidados intensivos usando las escalas ICU-CAM en pacientes con ventilación mecánica invasiva y CAM-S en pacientes sin ventilación mecánica invasiva ^10^^,^^11^. Además, se midió la fuerza muscular con la escala del *Medical Research Council* (MRC-SS) ^12^ al egreso de la unidad de cuidados intensivos y se registró diariamente el grado de movilidad con la escala IMS ^13^, donde los valores más altos indican una mayor movilidad.

Para estandarizar la medición con la escala de movilidad en unidad de cuidados intensivos (IMS) y MRC-SS, las fisioterapeutas encargadas de realizarlas y registrarlas recibieron una capacitación por parte del fisiatra. Como desenlace primario se consideró la reducción de la incidencia de debilidad adquirida en la unidad de cuidados intensivos al momento del egreso, que se determinó mediante la escala de fuerza muscular MRC-SS con un valor menor de 48 (en una escala que va desde 0 a 60) ^12^.

Los desenlaces secundarios incluyen: el nivel de movilidad al egreso de la unidad de cuidados intensivos según la IMS (valores de 0 a 10), el valor de fuerza muscular al egreso de la unidad de cuidados intensivos medido por la MRC-SS, la frecuencia y los días de *delirium* durante la hospitalización en la unidad de cuidados intensivos, los días transcurridos desde el ingreso a UCI hasta la primera sesión de fisioterapia, la frecuencia de sesiones de fisioterapia y terapia ocupacional por paciente en la unidad de cuidados intensivos, eventos adversos graves durante la movilización en la unidad de cuidados intensivos y mortalidad hospitalaria.

### Análisis estadístico

Se usó estadística descriptiva para las variables cuantitativas y estadística de resumen con medidas de tendencia central y de dispersión, de acuerdo con la distribución de las variables. Para las distribuciones no paramétricas, las variables se expresaron en medianas y rangos intercuartílicos, y para la comparación entre grupos se utilizó la prueba de rangos de Wilcoxon. Cuando la distribución fue normal, se reportaron las medias y las desviaciones estándar, y la comparación entre grupos se hizo con la prueba t de Student.

La prueba de normalidad para las variables numéricas fue la de Shapiro-Wilk. Para las variables cualitativas se aplicaron estadísticas de resumen como frecuencias relativas y la comparación entre grupos se realizó con prueba de ji al cuadrado o test exacto de Fisher, según la naturaleza de la variable y su distribución.

Se hizo análisis multivariado, tipo regresión lineal múltiple y logística, donde la variable dependiente fue, en el primer caso, el valor numérico de las escalas de fuerza muscular y, en el segundo, la variable de fuerza muscular categorizada como la presencia de debilidad muscular adquirida en unidad de cuidados intensivos. La selección de las variables de ingreso a la regresión logística binaria se hizo según el estudio de asociación de análisis univariado con un valor de p menor de 0,2 y otras variables clínicas exploradas como factores de riesgo, identificados en la revisión de la literatura. En el resultado de la regresión binaria se muestra la razón de probabilidades y el valor de p (significancia estadística cuando p < 0,05). Todos los análisis fueron realizados con Stata/IC 16™ (Stata Co., College Station, TX, USA).

## Resultados

**Figura 1:**
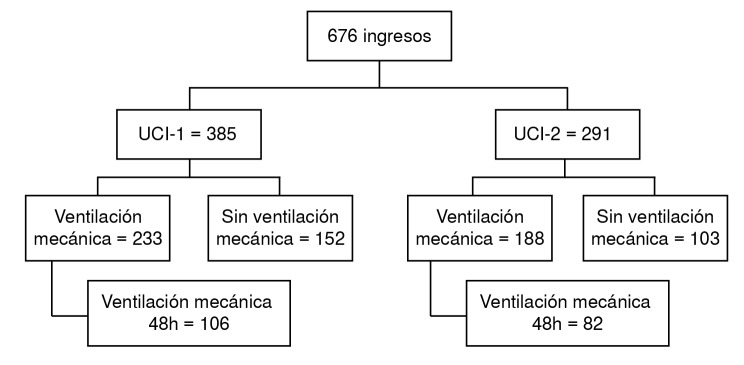
Algoritmo de ingreso

Durante el estudio, 676 pacientes fueron ingresados a las dos unidades de cuidados intensivos. De ellos, 188 tuvieron ventilación mecánica invasiva durante 48 horas o más (figura 1). En el cuadro 1 se presentan las variables demográficas y clínicas de los pacientes.

**Cuadro 1:** Datos demográficos

Variable	Grupo control	Grupo intervención	P
	(UCI-1)	(UCI-2)	
	n=106	n=82	
Edad, [mediana (RIC)]	56 (39-75)	56 (33-66)	0,83
Sexo masculino, [n (%)]	58 (55)	48 (59)	0,65
APACHE II, [mediana (RIC)]	19 (16-25)	21 (14-26)	0,80
Escala de Charlson, [mediana (RIC)]	2 (0-4)	3(0-5)	0,185
Comorbilidades [n (%)]	48 (45)	46 (56)	0,185
Diabetes mellitus	20 (18,7)	12(14,6)	0,705
EPOC	18 (16,8)	16(19,5)	0,705
Insuficiencia cardíaca congestiva	10 (9,4)	6(7,3)	0,793
Falla renal crónica	11 (10,4)	12(14,6)	0,381
Neoplasias sólidas	5 (4,7)	12(14,6)	0,022
Tipo de patología			0,369
Médica	70 (66)	46 (56)	
Quirúrgica	13 (12,3)	12(14,6)	0,664
Trauma	23 (21,7)	24 (29,3)	
Causas de ingreso a la unidad de cuidados intensivos			
Falla respiratoria aguda	34 (32,1)	29 (35,3)	
Alteración del estado de consciência	28 (26,4)	23 (28)	
Falla circulatoria (choque)	14(13,2)	8(9,8)	
Postoperatorio de cirugía mayor	7 (6,7)	8(9,8)	

RIC: rango intercuartílico; EPOC: enfermedad pulmonar obstructiva crónica

En el grupo de intervención se realizó mayor movilización temprana (97,6 % vs 55,7 %), iniciada en menos tiempo (dos días versus cuatro días); también se realizó un mayor número de sesiones por paciente (cuatro versus tres) (cuadro 2). En el 34,1 % de los casos, los pacientes del grupo de intervención realizaron ejercicios en un cicloergómetro, mientras que solo el 9,4 % de los pacientes del grupo control lo hicieron. La debilidad adquirida en unidad de cuidados intensivos al egreso de fue significativamente menor en el grupo de intervención (41 % versus 79 %), lo cual se correlaciona con una mayor fuerza muscular al egreso de la unidad de cuidados intensivos por MRC-SS (48 versus 36) y mayor movilidad al egreso de la unidad de cuidados intensivos (IMS 3,5 versus 2) (cuadro 2).

**Cuadro 2:** Intervenciones de movilidad y principales resultados

Variable	Control		Intervención	P
Fisioterapia, movilización temprana o ambas, [n (%)]	59	(55,7)	80 (97,6)	<0,00001
Días al inicio de la fisioterapia o movilidad, [mediana (RIC)]	4	(2-7)	2 (1-3)	<0,00001
Número de sesiones por paciente, [mediana (RIC)]	3	(2-6)	4 (3-8)	0,041
Número de sesiones de terapia ocupacional, [media (DE)]	0,2		0,5	0,059
Debilidad adquirida en UCI MRC-SS <48, [n/N (%)]	15/19	(79)	19/46(41)	0,003
Fuerza muscular al egreso, (escala MRC), [mediana (RIC)]	36	(36-40,5)	48 (36-48)	0,0045
Escala de movilidad al egreso: mediana (RIC)	2	(0-4)	3,5 (0-5)	0,0138
Escala de movilidad ≥ 4, [n (%)]	11	(23)	33 (50)	0,082

RIC: rango intercuartílico; n/N: evento en el que se realizó la escala MRC-SS; UCI: unidad de cuidados intensivos; DE: desviación estándar

La mediana de días libres de respiración mecánica asistida, unidad de cuidados intensivos y hospitalización a los 28 días fue mayor en el grupo de intervención. También se observó una disminución en la mortalidad general en el grupo de intervención en comparación con el grupo control (18,2 % versus 29 %), pero estas diferencias no fueron estadísticamente significativas (cuadro 3).

**Cuadro 3:** Resultados secundarios

Variable	Control	Intervención	p
Días libres de respiración mecánica asistida al día 28, [mediana (RIC)]	15 (0-24)	18 (0-23)	0,496
Días libres de UCI al día 28, [mediana (RIC)]	14 (0-21)	14,5 (0-21)	0,97
Días libres de hospitalización al día 28, [mediana (RIC)]	0 (0-12)	0 (0-11)	0,462
Días libres de hospitalización al día 28, [media (DE)]	5,3 (8)	5,9 (7,4)	0,462
Mortalidad general, [n (%)]	29 (27,3)	15 (18,2)	0,167

RIC: rango intercuartílico; DE: desviación estándar

Durante la movilización activa en el grupo de intervención, un paciente experimentó el evento adverso de desalojo de la sonda nasogástrica. Durante los seis meses de duración del estudio, un paciente del grupo de intervención y tres pacientes del grupo control fueron diagnosticados con neumonía asociada al respirador. La incidencia de *delirium* fue del 14,5 % en el grupo control y del 15,8 % en grupo de intervención, con un promedio de 2 y 1,8 días de duración, respectivamente. Un paciente de cada grupo fue remitido a una institución por ventilación mecánica invasiva crónica.

En el análisis multivariado, tipo regresión logística múltiple, para la variable categorizada de debilidad muscular (menor de 40 y mayor o igual a 48) se encontró una asociación significativa entre la disminución de la debilidad adquirida en la unidad de cuidados intensivos y la movilidad activa temprana (OR = 7,00, IC _95_ % = 3,6-13,7) (cuadro 4).

**Cuadro 4:** Análisis multivariado de la debilidad adquirida en la unidad de cuidados intensivos

	*Odds ratio*	Error estándar	z	p	IC95 %	
Sexo	0,75	0,26	-0,82	0,415	0,38	1,50
Edad	1,005	0,011	0,50	0,62	0,98	1,02
APACHE II	1,002	0,019	0,15	0,87	0,96	1,041
CHARLSON	1,05	0,087	0,66	0,51	0,90	1,24
Intervención	7,00	2,40	5,68	<0,0001	3,57	13,7
Constante	0,16	0,109	2,68	0,007	0,041	0,61

IC: intervalo de confianza

Regresión logística de la variable dependiente (debilidad adquirida en la unidad de cuidados intensivos definida como una escala de fuerza motora menor o igual a 48 al egreso de la unidad) y las independientes a probar: la intervención, la estilísticamente asociada -CHARLSON- y las variables clínicas de relevancia APACHE II, sexo y edad. Número de observaciones: 188. LR X^2 (^^4^ = 39,19; p=0,0000; log *likelihood* = -104,52; Pseudo R^2^: 0,157

## Discusión

En este estudio, por medio de la implementación de un protocolo multicomponente, se encontraron diferencias estadísticamente significativas a favor del grupo de intervención, tanto en alcanzar una mayor cantidad de movilizaciones activas en la unidad de cuidados intensivos (97,6 % versus 55,7 %), como en la disminución de la incidencia de la debilidad adquirida en la unidad de cuidados intensivos al egreso de la unidad (41 % versus 79 %); mejor movilidad al egreso de la unidad de cuidados intensivos (IMS: 3,5 versus 2) y mayor fuerza muscular al egreso de la unidad de cuidados intensivos (MRC-SS = 48 versus 36).

Nuestro hallazgo es similar a lo reportado en la revisión sistemática y metanálisis de Wang y colaboradores ^14^, donde encontraron que la movilidad temprana disminuye la debilidad adquirida en la unidad de cuidados intensivos (RR = 0,49, IC _95%_ = 0,32-0,74, p=0,0008]) y mejora el puntaje de la MRC-SS. En otro metanálisis más reciente, al comparar movilidad temprana sistemática versus temprana estándar, no encontraron diferencias significativas tanto en la debilidad adquirida en la unidad de cuidados intensivos (RR = 0,90; IC _95%_ = 0,63-1,27), como en la MRC-SS (mediana = 5,8; IC _95%_ = -1,4-13). Igualmente, al comparar movilidad temprana sistemática versus movilidad tardía para la debilidad adquirida (RR = 0,62; IC _95%_ = 0,381,03) ^15^.

Este estudio también halló una incidencia baja en la presentación del *delirium,* y las diferencias entre los grupos de intervención y control fueron mínimas. Esto coincide con los resultados de la revisión sistemática y el metanálisis de Wang y colaboradores, quienes no encontraron una reducción significativa en la incidencia de *delirium* (RR = 0,52, IC _95%_ = 0,19-1,44, p=0,21) ^14^.

Los resultados contradictorios de los estudios de movilidad temprana ^5^^,^^11^^,^^16^^-^^18^ podrían ser parcialmente explicados por la falta de uniformidad en las definiciones. En la revisión sistemática realizada por Menges y colaboradores ^15^, la definición de movilización temprana fue cualquier terapia física u ocupacional para la activación muscular aplicada sistemáticamente dentro de los primeros siete días de ingreso a la unidad de cuidados intensivos, mientras que la utilizada en el metanálisis de Wang y colaboradores ^14^ fue la iniciada en un tiempo de ventilación mecánica invasiva menor de 72 horas. A diferencia de los estudios que iniciaron tardíamente (más de 48 horas con respiración mecánica asistida), la mayoría de los estudios de movilidad temprana (menos de 48 horas) han demostrado mejor pronóstico en cuanto a recuperación de la independencia funcional al egreso del hospital, menos *delirium* y más días libres de ventilación mecánica invasiva ^19^^-^^21^.

En un ensayo clínico aleatorizado reciente ^22^, la movilidad activa temprana no logró disminuir de manera estadísticamente significativa la mortalidad. En este estudio, la movilidad activa se realizó de manera aislada, a diferencia de lo propuesto por la SCCM en *The ICU Liberation ABDCE Bundle,* donde la movilidad activa temprana debe ser aplicada en conjunto con las otras medidas, ya que tiene una relación de interdependencia con la sedación, el dolor no controlado y el *delirium*^7^^,^^8^^,^^23^.

En el presente estudio, además de aplicar unos criterios de inclusión más parecidos a los estudios del metanálisis de Wang, se implementaron varias estrategias al tiempo, cinco de las seis recomendadas por la intervención multicomponente *ABCDEF Bundle*^7^. Con esta estrategia se han obtenido mejores resultados en 15.000 pacientes con la aplicación de un número mayor de componentes en resultados, como mayor supervivencia, menos días de ventilación mecánica y menos *delirium* y coma ^7^.

Las fortalezas de este estudio incluyen su diseño con controles concurrentes, la base en un modelo de atención multicomponente y la creación de un grupo multiprofesional para la implementación del protocolo que permitió una mayor coordinación y eficacia en la aplicación de este.

Como debilidades se reconocen la falta de aleatorización, su realización en un solo centro y la imposibilidad de limitar la movilidad de los médicos tratantes entre las dos unifdades de cuidados intensivos del estudio. Otra debilidad podría ser la falta de evaluación del impacto económico y la viabilidad de la implementación en otros centros. Finalmente, la ausencia de información sobre el pronóstico a largo plazo puede limitar la comprensión completa de los efectos de la intervención en el paciente y su posterior calidad de vida.

En conclusión, la implementación de un modelo de atención multicomponente protocolizada, basado en el *ABCDEF Bundle* y que incluyó: movilidad activa temprana, uso limitado de sedantes, analgesia adecuada, medidas no farmacológicas para prevenir el *delirium,* estimulación cognitiva y empoderamiento de familiares, disminuyó de manera significativa la debilidad adquirida en la unidad de cuidados intensivos al momento del egreso.

## Archivos suplementarios

### Anexo 1.

**Table:** 

Modelo estándar (control)	Modelo protocolizado (intervención)
Modelo que venía siendo aplicado en todas las unidades de cuidados intensivos antes de julio de 2018.	
Ronda multiprofesional	Ronda multiprofesional
No se realizaban.	Médico intensivista no tratante, intensivista tratante, fisiatra, fisioterapeuta, terapeuta respiratorio, químico farmacéutico y enfermería. Se realizaban de lunes a viernes a las 09:00 a. m.
Evaluación, prevención y manejo del dolor	Evaluación, prevención y manejo del dolor
En todos los pacientes del hospital se hace evaluación con escala de dolor y se aplica un protocolo para la prevención y manejo óptimo del dolor.	Además del manejo estándar, en la ronda multiprofesional diaria se hacía reevaluación del control adecuado del dolor.
Sedación adecuada	Sedación adecuada
El intensivista tratante utilizaba midazolam en infusión, según su criterio. El nivel de sedación (RASS) lo decidía el médico tratante.	En la ronda se recomendaba al intensivista tratante evitar el uso de midazolam en infusión. En caso de ser necesario, se utilizaba en bolos. Se trataba de mantener a los pacientes despiertos (RASS 0-1) durante el día en caso de que no tuvieran contraindicación.
Evaluar, prevenir y tratar el *delirium*	Evaluar, prevenir y tratar el *delirium*
En todos los pacientes de las unidades de cuidados intensivos se aplican las escalas para la evaluación de la confusión ICU-CAM o CAM-S por enfermeras, dos veces al día. En caso de ser positivos para *delirium*, los pacientes eran evaluados por psiquiatría y se iniciaba tratamiento farmacológico. No se realizaban medidas preventivas no farmacológicas.	En todos los pacientes de las unidades de cuidados intensivos se aplicaban las escalas ICU-CAM o CAM S por enfermeras, dos veces al día. En caso de ser positivos para *delirium*, los pacientes eran evaluados por psiquiatría y se iniciaba tratamiento farmacológico. Se realizaban medidas preventivas no farmacológicas (estimulación cognitiva, orientación espacial y temporal por parte del personal tratante y la familia).
Movilidad activa temprana	Movilidad activa temprana
Se iniciaba según el criterio del intensivista tratante. El tipo de ejercicio y la movilización era definidos por la fisioterapeuta sin seguir ningún protocolo. La fisioterapeuta estaba a cargo de dos unidades de cuidados intensivos (28 pacientes).	Se iniciaba en las primeras 48 horas de ingreso a la unidad de cuidados intensivos, lo cual era verificado en la ronda multiprofesional. La movilidad se realizaba, de acuerdo con el protocolo descrito abajo. La fisioterapeuta estaba a cargo de una unidad de cuidados intensivos (12 pacientes).
Familia	Familia
No se solicitaban compromisos a los familiares. Acompañamiento familiar durante las 16 horas día.	Compromiso de familia con los ejercicios físicos enseñados por la fisioterapeuta, las medidas para prevenir el *delirium* en el transcurso del día y acompañamiento en las caminatas y paseos fuera de la unidad de cuidados intensivos. Acompañamiento familiar durante las 24 horas del día.

### Protocolo de movilización temprana en pacientes de cuidados intensivos

#### Cuidados de enfermería en la implementación de la movilización temprana en pacientes

- Verificar que los pacientes que ingresen a la unidad de cuidados intensivos tengan la interconsulta con fisioterapia.
- La movilidad se debe iniciar en las primeras 48 horas de hospitalización en la unidad de cuidados intensivos, si no hay contraindicaciones. Los criterios de inclusión para el inicio de la movilización se deben verificar en compañía del médico intensivista y el grupo de fisioterapia.
- Realizar seguimiento de los pacientes a los que se les aplican los ejercicios de movilización y las posibles complicaciones que puedan presentar.
- Verificar en el plan de cuidados de enfermería la realización de la meta de cuidado por parte del grupo de fisioterapia.
- Verificar que las recomendaciones brindadas por parte del grupo de fisioterapia sean realizadas por el grupo de enfermería y el cuidador principal.
- Incentivar al cuidador principal del paciente y su familia, la participación en la realización de los ejercicios propuestos por parte del grupo de fisioterapia.
- Brindar al paciente medidas de confort y control del dolor que le permitan observar el plan de ejercicios implementado por parte del grupo de fisioterapia.

### Contraindicaciones de movilidad activa

- Dosis significativas de vasopresores (norepinefrina mayor a 0,15 µg/ kg/minuto) para mantener la presión arterial media mayor a 60 mm Hg
- Paciente con respiración mecánica asistida que requiera una fracción de oxígeno inspirado mayor a 0,6 o presión positiva al final de la espiración mayor de 10 mm Hg, o que tenga un deterioro agudo de la insuficiencia respiratoria.
- Evento neurológico agudo (accidente cerebrovascular, hemorragia subaracnoidea, hemorragia intracraneal) en las primeras 24 horas del paciente. La movilidad activa se debe iniciar después de 24 horas y en caso de hemorragia subaracnoidea espontánea, 12 horas después de la exclusión.
- Edema cerebral
- Estatus epiléptico
- Muerte cerebral
- Proceso hemorrágico activo
- Paciente con columna vertebral inestable o fracturas de extremidades
- Paciente con un pronóstico grave de final de vida, transferido a cuidados de confort
- Paciente con abdomen abierto (riesgo de dehiscencia)
- Primeras 72 horas de nueva trombosis venosa profunda (solo aplicable para la rehabilitación de la extremidad afectada y para la deambulación)
- Catéter arteria femoral

### Contraindicaciones para la movilidad pasiva

- Pacientes con orden de mínima manipulación

### Lista de chequeo antes de iniciar la movilización de pacientes en la unidad de cuidados intensivos

Verificar:

- Cumplimiento de criterios de inclusión
- Sin contraindicaciones
- Aprobación del médico tratante
- Se avisó a todo el personal necesario de la hora de inicio de la movilización.
- Verificar que todo esté listo antes de movilizar.
- Desenredar los cables y dejarlos holgados, y retirar los que no se necesitan.
- Dejar el espacio necesario por el lado de la cama por donde se sentará el paciente (lado donde tenga el catéter y preferir el siguiente orden de importancia: central, arterial y periférico).
- Traer el monitor de transporte, si va a caminar fuera de la habitación o la unidad de cuidados intensivos.
- Traer el maletín de transporte, si va a caminar fuera de la unidad de cuidados intensivos.
- Traer silla de ruedas, si va a caminar fuera de la habitación o fuera de la unidad de cuidados intensivos.

### Procedimiento antes de cada sesión de movilidad

- El médico se encargará de ajustar la sedación.
- El personal de enfermería debe encargarse de suministrar los medicamentos para el dolor (30 minutos antes), si el paciente lo requiere.
- Se debe realizar limpieza de secreciones, ajustar los parámetros de la respiración mecánica asistida y disminuir el trabajo respiratorio 30 minutos antes de la terapia respiratoria.
- Antes de la movilidad del paciente, el fisioterapeuta deberá aplicar cada día la escala de movilidad en la unidad de cuidados intensivos.
- Una vez aplicada la escala de movilidad, el fisioterapeuta deberá realizar con cada paciente, según las condiciones clínicas de cada uno discutidas con el grupo de médicos y enfermeros, ejercicios de movilidad pasiva, fisioterapia asistida activa, libre, resistida o ambas de sus extremidades para mejorar la fuerza muscular y la movilidad de manera progresiva.
- El fisioterapeuta deberá realizar la meta de cuidado y definir su plan de movilidad que será consignado en el plan de cuidado integral en la historia clínica.
- El último día de hospitalización en la unidad de cuidados intensivos se debe realizar la evaluación de fuerza muscular con la escala MRC-SS. Esta actividad la realiza el fisioterapeuta.
- Al ingreso y al egreso de la unidad de cuidados intensivos se debe aplicar la escala de funcionalidad.

### Consideraciones para la fisioterapeuta con cada sesión de rehabilitación física o movilidad en la unidad de cuidados intensivos

- Determinar que el nivel de actividad sea terapéutico.
- Programar un tiempo para trabajar en la actividad física con el paciente, la familia del paciente, la enfermera y el terapeuta respiratorio. Determinar si la sedación debe ser suspendida.
- Evaluar y controlar el dolor del paciente antes, durante y después de la movilidad.
- Optimizar el trabajo de respiración y el nivel de alerta del paciente para que el tratamiento sea beneficioso.
- Crear actividades que estén orientadas a los objetivos del paciente.
- No demorar ni retrasar la actividad física y la rehabilitación porque el paciente debe ser extubado ese día.
- No demorar ni retrasar la actividad física debido a la agitación, si la enfermera y el terapeuta pueden manejarla con seguridad. En pacientes que estén agitados o que experimenten un pensamiento desorganizado y delirio, una tarea enfocada brinda la oportunidad de reorientar la conversación.

### Personal necesario para las transferencias

- Transferencia de la cama a la silla sin caminar, de supino a sedente, y de sedente a bípedo, en pacientes con respiración mecánica asistida y catéter venoso central
- *Personal responsable:* fisioterapeuta, terapeuta respiratorio, auxiliar de enfermería o enfermera jefe
- Caminata dentro de la UCI
- *Personal responsable:* fisioterapeuta, terapeuta respiratorio, auxiliar de enfermería o enfermera jefe y familiar (lleva la silla de ruedas)
- Caminata fuera de la la unidad de cuidados intensivos.
- *Personal responsable:* fisioterapeuta, terapeuta respiratorio, enfermera jefe o auxiliar de enfermería, médico y familiar

### Responsabilidades de vigilancia para la movilidad

- Terapeuta respiratorio en pacientes con respiración mecánica asistida: revisar vía aérea, monitor y respirador en caso de tubo orotraqueal, traqueostomía o respiración mecánica no asistida.
- Enfermera jefe: catéteres vasculares centrales
- Auxiliar de enfermería: catéteres venosos, periféricos, vesicales, sonda a tórax, bombas de infusión
- Familiar o acompañante: silla de ruedas
- Fisioterapeuta: movimientos del paciente y verificación de su estado clínico
- Médico: estado clínico del paciente y verificación de todo

### Pasos para la movilidad en la unidad de cuidados intensivos

- Paso 1: sentar al paciente en la cama; mirar al paciente, el monitor y las líneas.
- Paso 2: sentar al paciente en el borde de la cama, evaluar el dolor y la presión arterial ortostática.
- Paso 3: asistir al paciente para pasar de sentado a de pie. Si no puede caminar, sentarlo en la silla.
- Paso 4: la deambulación debe ser con la ayuda de un caminador y se debe mantener una silla cerca del paciente. Contar con apoyo de familiares, asistentes, voluntarios y estudiantes para empujar la silla y los atriles.
- Paso 5: sentar al paciente cuando sea necesario.

### Detenga y descanse al paciente si:

- No responde.
- Tiene aspecto fatigado y pálido.
- Tiene una frecuencia respiratoria consistentemente mayor a 10 latidos por minuto por encima de la línea de base.
- Pérdida del equilibrio
- Disminución de la capacidad para caminar
- Diaforesis

### Riesgos

- Deterioro del estado clínico del paciente (taquicardia, desaturación)
- Desplazamientos de tubos, sondas o catéteres
- Caídas

### Consideraciones en pacientes en aislamiento

1. Si el paciente está aislado por infección por SARS-CoV-2, se debe realizar movilización por parte de fisioterapia y personal de enfermería dentro de la habitación, sin acompañante, hasta que cumpla el periodo de aislamiento que son 15 días. Una vez cumplido este periodo se puede iniciar el programa de movilización establecido.
2. Si el paciente tiene aislamiento de contacto, puede salir de la habitación en compañía del personal que se requiera según su estado, con los elementos de protección personal evitando el contacto con superficies y, de ser así, se debe realizar limpieza de estas superficies de acuerdo con la guía de limpieza y desinfección.
3. Si son pacientes con aislamiento de gotas o aislamiento aéreo, se debe realizar la movilización dentro de la habitación o según la consideración del médico tratante.

### Tabla anexo 1. Niveles de actividad según la condición clínica (adaptado del consenso de expertos y las recomendaciones en los criterios de seguridad para la movilización activa)

Definición de códigos de colores:

**Figure:**
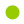

Bajo riesgo de un evento adverso. Proceder como de costumbre de acuerdo con los protocolos y procedimientos de cada UCI.

**Figure:**
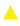

El riesgo potencial y las consecuencias de un evento adverso son más altos que el verde, pero pueden verse compensados por los beneficios de la movilización. Las precauciones o contraindicaciones deben aclararse antes de cualquier episodio de movilización. Si se moviliza, se debe considerar hacerlo de manera gradual y con cautela.

**Figure:**
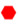

Riesgo potencial significativo o consecuencias de un evento adverso. La movilización activa no debe ocurrir a menos que esté específicamente autorizada por el médico especialista, tratante, de cuidados intensivos; en consulta con el fisioterapeuta y el personal de enfermería ^8^^,^^9^.

**Table:** 

Parámetros respiratorios	Ejercicios en cama	Ejercicios fuera de la cama
Fracción de oxígeno inspirado ≤ 0,6	**Figure:** 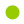	**Figure:** 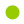
Fracción de oxígeno inspirado > 0,6	**Figure:** 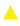	**Figure:** 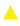

Precauciones de saturación de oxígeno (SpO_2_)	Ejercicios en cama	Ejercicios fuera de la cama
≥ 90 %	**Figure:** 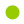	**Figure:** 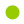
< 90 %	**Figure:** 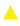	**Figure:** 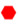

Frecuencia respiratoria	Ejercicios en cama	Ejercicios fuera de la cama
≤ 30 respiraciones por minuto	**Figure:** 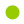	**Figure:** 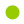
> 30 respiraciones por minuto	**Figure:** 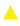	**Figure:** 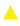

Ventilación	Ejercicios en cama	Ejercicios fuera de la cama
Modo ventilatorio de oscilación de alta frecuencia	**Figure:** 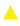	**Figure:** 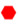

Presión positiva al final de la espiración	Ejercicios en cama	Ejercicios fuera de la cama
≤ 10 cm H_2_0	**Figure:** 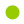	**Figure:** 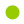
> 10 cm H_2_O	**Figure:** 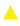	**Figure:** 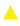
Asincronía con el respirador	**Figure:** 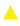	**Figure:** 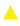

Terapias de rescate	Ejercicios en cama	Ejercicios fuera de la cama
Óxido nítrico	**Figure:** 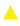	**Figure:** 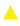
Prostaciclina	**Figure:** 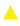	**Figure:** 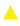
Paciente en posición prona	**Figure:** 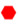	**Figure:** 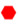
		
Consideraciones cardiovasculares		
Presión sanguínea	Ejercicios en cama	Ejercicios fuera de la cama
Terapia antihipertensiva venosa por emergencia hipertensiva	**Figure:** 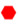	**Figure:** 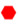

Presión arterial media	Ejercicios en cama	Ejercicios fuera de la cama
Debajo del rango objetivo y causante de síntomas	**Figure:** 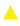	**Figure:** 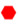
Debajo del rango objetivo a pesar de ser soportado(vasoactivo, mecánico o ambas).	**Figure:** 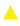	**Figure:** 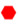
Mayor que el límite inferior mientras no reciba soporte o soporte de bajo nivel (norepinefrina ≤ 0,15 mg/kg/ minuto)	**Figure:** 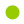	**Figure:** 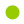
Mayor que el límite inferior del rango objetivo, mientras reciba soporte de nivel moderado (norepinefrina ≥ 0,15 y < 0,2 mg/kg/minuto).	**Figure:** 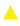	**Figure:** 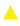
Mayor que el límite inferior del rango objetivo, con soporte de alto nivel (norepinefrina > 0,2 mg/kg/ minuto)	**Figure:** 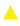	**Figure:** 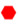
Sospecha de hipertensión pulmonar grave	**Figure:** 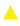	**Figure:** 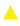

Arritmia cardíaca	Ejercicios en cama	Ejercicios fuera de la cama
Bradicardia		
Requiere tratamiento farmacológico (por ejemplo, isoproterenol) o inserción de marcapasos de emergencia a demanda.	**Figure:** 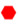	**Figure:** 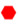
No requiere tratamiento farmacológico ni una inserción de marcapasos de emergencia a demanda.	**Figure:** 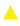	**Figure:** 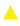
Marcapasos transvenoso o epicárdico	Ejercicios en cama	Ejercicios fuera de la cama
Ritmo dependiente	**Figure:** 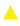	**Figure:** 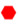
Ritmo de base estable	**Figure:** 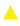	**Figure:** 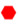

Cualquier taquiarritmia estable	Ejercicios en cama	Ejercicios fuera de la cama
Frecuencia ventricular: > 150 latidos por minuto	**Figure:** 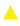	**Figure:** 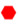
Frecuencia ventricular: 120-150 latidos por minuto	**Figure:** 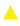	**Figure:** 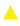
Cualquier taquiarritmia con frecuencia ventricular <120 latidos por minuto	**Figure:** 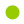	**Figure:** 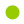

Dispositivos	Ejercicios en cama	Ejercicios fuera de la cama
Balón de contrapulsación femoral	**Figure:** 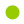	**Figure:** 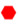

Oxigenación por membrana extracorpórea	Ejercicios en cama	Ejercicios fuera de la cama
Femoral o subclavio	**Figure:** 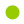	**Figure:** 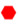
Cánula doble, luz bicaval simple, insertado en una vena central	**Figure:** 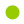	**Figure:** 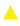
Dispositivo de asistencia ventricular	**Figure:** 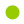	**Figure:** 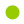
Catéter de arteria pulmonar u otro dispositivo de monitoreo de gasto cardíaco	**Figure:** 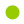	**Figure:** 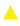

Otras consideraciones cardiovasculares	Ejercicios en cama	Ejercicios fuera de la cama
Choque de cualquier causa con lactato > 4 mmol/L	**Figure:** 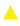	**Figure:** 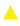
Trombosis venosa profunda o tromboembolismo pulmonar agudos, conocidos o con sospecha en las primeras 72 horas de anticoagulación.	**Figure:** 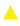	**Figure:** 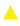
Estenosis aórtica severa conocida o con sospecha Isquemia cardíaca (definido como dolor torácico continúo y/o cambios dinámicos en el electrocardiograma)	**Figure:** 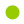	**Figure:** 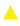
	**Figure:** 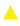	**Figure:** 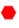
Otras consideraciones		
Quirúrgicas	Ejercicios en cama	Ejercicios fuera de la cama
Fractura mayor inestable/no estabilizada: Pelvis Columna Extremidad inferior /hueso largo	**Figure:** 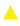	**Figure:** 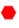
Gran herida quirúrgica abierta: Gran herida quirúrgica abierta/esternón Abdomen	**Figure:** 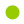	**Figure:** 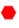

Médicas	Ejercicios en cama	Ejercicios fuera de la cama
Hemorragia activa (conocida) no controlada	**Figure:** 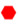	**Figure:** 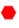
Sospecha de sangrado activo o riesgo de sangrado mayor	**Figure:** 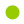	**Figure:** 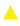
Paciente febril con una temperatura que excede el máximo aceptable en manejo activo de enfriamiento físico o farmacológico	**Figure:** 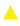	**Figure:** 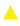
Manejo activo de hipotermia	**Figure:** 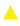	**Figure:** 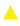
Otras consideraciones	Ejercicios en cama	Ejercicios fuera de la cama
Debilidad adquirida en la UCI	**Figure:** 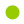	**Figure:** 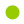
Terapia de reemplazo renal continuo (sin incluir catéteres de diálisis femoral).	**Figure:** 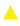	**Figure:** 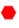
Catéteres femorales venosos	**Figure:** 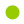	**Figure:** 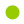
Catéteres arteriales femorales (introductor y línea para la monitorización de la presión arterial)	**Figure:** 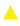	**Figure:** 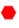
Otro tipo de dispositivos y accesorios:		
Sonda nasogástrica		
Catéter venoso central		
Sonda pleural	**Figure:** 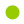	**Figure:** 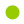
Drenaje de heridas		
Catéter intercostal		
Sonda vesical		
		
Consideraciones neurológicas		
Nivel de conciencia	Ejercicios en cama	Ejercicios fuera de la cama
Paciente somnoliento, tranquilo o inquieto (RASS = -1 o 1)	**Figure:** 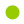	**Figure:** 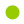
Paciente ligeramente sedado o agitado (RASS = -2 o 2)	**Figure:** 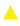	**Figure:** 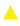
Paciente que no despierta al llamado o bajo sedación profunda (RASS < -2)	**Figure:** 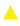	**Figure:** 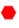
Paciente muy agitado o combativo (RASS > 2).	**Figure:** 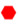	**Figure:** 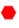
		
*Delirium*	Ejercicios en cama	Ejercicios fuera de la cama
Escala de *delirium* (ICU-CAM)	**Figure:** 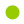	**Figure:** 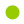
Escala de *delirium* y el paciente es capaz de seguir comandos simples	**Figure:** 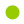	**Figure:** 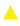
Escala de *delirium* y el paciente no puede seguir comandos	**Figure:** 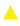	**Figure:** 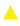
		
Presión intracraneal	Ejercicios en cama	Ejercicios fuera de la cama
Hiperpresión intracraneal en manejo activo o fuera del rango deseado	**Figure:** 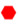	**Figure:** 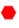
Monitorización de la presión intracraneal sin tratamiento activo de la hipertensión intracraneal.	**Figure:** 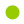	**Figure:** 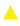
		
Presión intracraneal	Ejercicios en cama	Ejercicios fuera de la cama
Craniectomía	**Figure:** 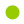	**Figure:** 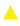
Drenaje lumbar abierto (sin clamp)	**Figure:** 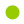	**Figure:** 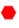
Drenaje subgaleal	**Figure:** 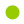	**Figure:** 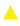
Precauciones espinales (previo lavado o fijación)	**Figure:** 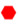	**Figure:** 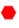
Lesión de la médula espinal	**Figure:** 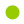	**Figure:** 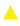
Hemorragia subaracnoidea con aneurisma no excluido	**Figure:** 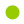	**Figure:** 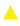
Vasoespasmo posaneurisma excluido	**Figure:** 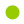	**Figure:** 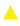
Convulsiones no controladas	**Figure:** 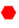	**Figure:** 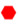
